# The Impact of *Moringa oleifera* Supplementation on Anemia and other Variables during Pregnancy and Breastfeeding: A Narrative Review

**DOI:** 10.3390/nu15122674

**Published:** 2023-06-08

**Authors:** Rosita Rotella, Jose M. Soriano, Agustín Llopis-González, María Morales-Suarez-Varela

**Affiliations:** 1 Research Group in Social and Nutritional Epidemiology, Pharmacoepidemiology and Public Health, Department of Preventive Medicine and Public Health, Food Sciences, Toxicology and Forensic Medicine, Faculty of Pharmacy, Universitat de València, Av. Vicent Andrés Estelles s/n, 46100 Burjassot, Spain; rotella@alumni.uv.es (R.R.); agustin.llopis@uv.es (A.L.-G.); 2 Observatory of Nutrition and Food Safety for Developing Countries, Food & Health Lab, Institute of Materials Science, University of Valencia, Carrer Catedrático Agustín Escardino 9, 46980 Paterna, Spain; jose.soriano@uv.es; 3 Joint Research Unit on Endocrinology, Nutrition and Clinical Dietetics, University of Valencia-Health Research Institute La Fe, Avda. Fernando Abril Martorell, 106, 46026 Valencia, Spain; 4 Biomedical Research Center in Epidemiology and Public Health Network (CIBERESP), Carlos III Health Institute, Av. Monforte de Lemos 3-5, Pabellón 11, Planta 0, 28029 Madrid, Spain

**Keywords:** *Moringa oleifera*, pregnancy, breastfeeding, supplements or products with *Moringa*, natural galactagogues, malnutrition, developing countries

## Abstract

*Moringa* is a plant commonly used for its medical properties. However, studies have shown contradictory results. The aim of this review is to evaluate the possible association between the use of *Moringa* during pregnancy and breastfeeding in relation to the health status of both the mother and the baby. A search of the PubMed and EMBASE databases on the literature published during the period 2018–2023 was conducted up until March 2023. The population/exposure/comparison/outcome (PECO) approach was used to select studies on pregnant women, mother–child pairs, and the use of *Moringa*. Out of the 85 studies initially identified, 67 were excluded, leaving 18 for full-text evaluation. After assessment, 12 were finally included in the review. In the articles included in this work, *Moringa* is administered during pregnancy or in the postnatal period in the form of leaf powder (MOLP), as a leaf extract (MLE), as an ingredient associated with other supplements or in preparations. It appears to influence several variables during pregnancy and in the postnatal period such as the mother’s haematochemical profile, milk production, the child’s socio-personal development and the incidence of morbidity during the first 6 months of life. None of the studies analysed reported contraindications to the use of the supplement during pregnancy and lactation.

## 1. Introduction

Pregnancy is a state linked with considerable physiological changes resulting in various ailments and medical conditions, including nausea, vomiting, gastric disturbance, and respiratory illness. The mother’s diet and lifestyle before and during pregnancy and lactation constitute a determining factor in the infant’s health that can project on the wellbeing of the child and future adult [1].

Breast milk is the best food for babies becayse it is safe and clean, and contains antibodies that protect them against common illnesses. It also contains helpful nutrients and energy for babies, especially in the first month of life. Breastfeeding provides physiological and health-relted benefits for both the mother and the baby. The World Health Organization (WHO) and the United Nations International Children’s Emergency Fund (UNICEF) recommend that the baby be breastfed within the first hour and exclusively for the first 6 months of life. WHO actively promotes breastfeeding as the best source of nourishment for infants and young children and has set the rate of exclusive breastfeeding for the first 6 months as being up to at least 50% by the year 2025 [2].

Nutritional deficiencies are a significant health concern in developing countries and result in severe health consequences such as delayed physical and mental growth. *Moringa oleifera*, a highly nutritious plant grown in tropical regions of developing countries, has the potential to combat these deficiencies [3]. *Moringa* is well-known for its medicinal properties, with traditional use during pregnancy and breastfeeding to promote fetal growth and alleviate symptoms such as nausea, vomiting, and constipation [4]. *M. oleifera* Lam., belonging to the *Moringaceae* family, is a highly valuable plant with numerous applications in food, medicine, and industry. It has the remarkable ability to thrive in both humid tropical regions and hot, arid countries, making it adaptable to a wide range of environments, including barren or arid soils [5]. Originally native to the Indian and African continents, *Moringa oleifera* is now commercially cultivated in various parts of the world, including India, Africa, South and Central America, Mexico, Hawaii, Asia, and Southeast Asia. The extensive studies conducted on *Moringa* have not reported any adverse effects on human health [6]. However, it is important to note that the nutritional composition of this plant can vary significantly based on genetic factors, environmental conditions, and growing practices [7]. Therefore, the specific nutritional values of *Moringa* may differ depending on these variables.

*Moringa* leaves are an excellent source of essential proteins, vitamins, and minerals including iron, calcium, and vitamin C, which are crucial for both maternal and fetal health [8]. *Moringa* leaves exhibit a notable nutritional profile, with their estimated average values indicating that their protein, fiber, fat, and ash content range between 24.66–26.79 g/100 g, 18.67–20.99 g/100 g, 4.98–16.90 g/100 g, and 7.92–11.18 g/100 g, respectively. Furthermore, these leaves are recognized a valuable sources of essential amino acids, supplying approximately 43% of lysine, tryptophan, methionine, and cystine, and they are particularly abundant with regard to valine and leucine [9]. Additionally, *Moringa* leaves are rich in micronutrients. They exhibit high concentrations of iron (97.9 µg/g dried leaf), carotenoids (17.6–39.6 mg/100 g dried leaf), dietary fiber, B vitamins, vitamin C, calcium, and other essential nutrients, all of which have good bioavailability [10] However, there is limited scientific evidence to support the use of *Moringa* in these contexts, and it is vital to consult with healthcare providers before taking any supplements or herbal remedies during pregnancy or while breastfeeding [11]. *Moringa* has also been traditionally used to increase milk production and improve the quality of breast milk [12]. Although some studies suggest that *Moringa* may interact with certain medications or have adverse effects on blood sugar levels [13], more research is needed to evaluate its safety and efficacy.

Given the wide distribution of *M. oleifera* trees in areas with a high prevalence of malnutrition, understanding the potential benefits and risks of *Moringa* as a nutritional intervention during pregnancy and lactation is crucial for improving maternal and child health outcomes. This review aims to assess the most recent studies regarding the benefits of using *Moringa* as a dietary supplement during pregnancy and breastfeeding.

## 2. Materials and Methods

We conducted a systematic review to identify all studies investigating the association between *Moringa* intake and pregnancy, healthy children, and breastfeeding. The search was performed in the PUBMED and EMBASE databases using a uniform search strategy over a period ranging from 1 January 2018 to 31 March 2023. We applied no restrictions on the article type, but only considered human research published between 2018 and 2023. The following keywords were used for the search:

- (Moringa) AND (pregnancy)
- (‘Moringa’ OR moringa) AND (‘Pregnancy’ OR pregnancy)
- (Moringa) AND (breastfeeding)
- (‘Moringa’ OR moringa) AND (‘Breastfeeding’ OR breastfeeding)

We followed the population/exposure/comparison/outcome (PECO) framework for inclusion criteria, as recommended by the Cochrane Handbook for Systematic Reviews of Interventions. Studies meeting the following criteria were considered to be eligible for inclusion:

- Population (P): pregnant women, mother–child pairs, and breastfeeding women.
- Exposure (E): *Moringa* intake.
- Comparison (C): pregnant women not consuming *Moringa*.
- Study design (S): cross-sectional studies, case–control studies, cohort studies, propensity matching studies, reviews, and experimental studies.

The inclusion criteria for this review were as follows: (i) publication years: 2018–2023; (ii) Study type: human research studies comparing *Moringa* versus other micronutrient supplements or placebo; (iii) language: English; and (iv) article length: full-length articles.

We excluded investigations without outcomes related to pregnancy, healthy children, and breastfeeding. The following criteria were applied for exclusion as are animal studies, reviews, non-experimental studies, in vitro studies, case studies, protocols and abstracts.

**Figure 1:**
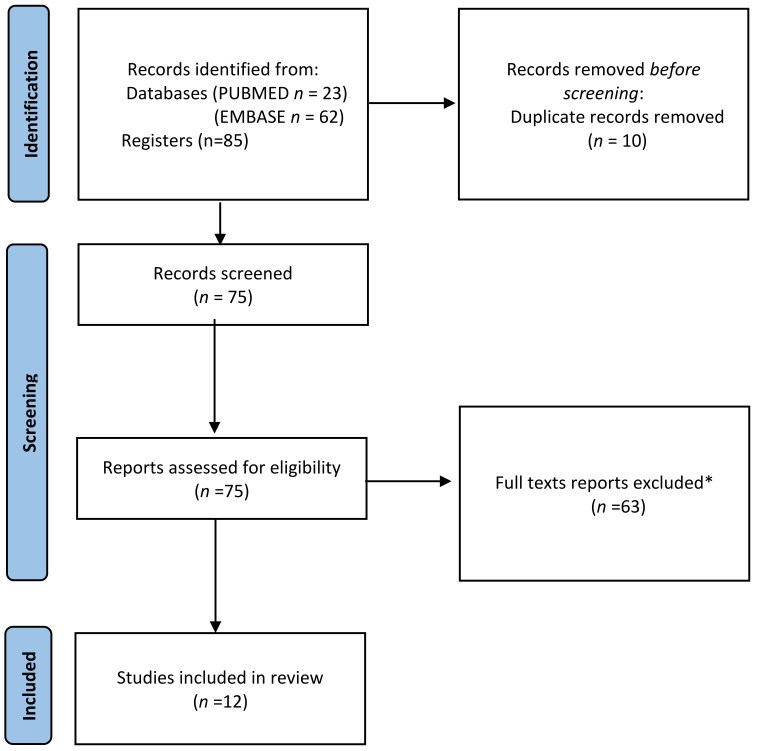
PRISMA (Preferred Reporting Items for Systematic Reviews and Meta-Analyses) flow diagram for studies retrieved through the searching and selection process. * The reasons for the exclusion of articles were reflected in Section 2.

The flow diagram of the study selection process review is presented in Figure 1 according to the PRISMA 2020 statement [14]. Two reviewers (R.R. and M.M.-S.-V.) with experience in reviews independently screened the titles, abstracts and full texts for eligibility, assessed study generalizability and collected data from each article. The third paired reviewer (J.M.S.) helped to resolve any disagreements. For data analysis, we summarized the main findings of each study by classifying them by year, country, target population, study design, interventions, considered variables, main findings and conclusion.

## 3. Results

### 3.1. Moringa and Pregnancy

Iron deficiency affects more than 2 billion people worldwide, with a high incidence in middle- and low-income countries. Iron deficiency anaemia (IDA) is a form of anaemia that accounts for 30–50% of all cases [10]. Due to the high prevalence of maternal IDA in developing countries [15] and the high iron content of *Moringa*, most studies available in the literature have focused on monitoring the effects of *Moringa* supplementation on anaemia during pregnancy and the postpartum period. Anaemia is one of the most frequent complications during pregnancy. This is due to the increase in plasma volume during pregnancy, which leads to a physiological decrease in haemoglobin concentration. Iron supplementation is known to increase the red cell mass, thereby increasing the oxygen-carrying capacity of pregnant woman [16]. *Moringa* supplementation has also been shown to increase haemoglobin levels, as well as other haematological parameters such as haematocrit, MCH, MCHC, and MCV values [9]. These benefits can be attributed to the presence of iron in the *Moringa* supplement. Therefore, *Moringa* can be considered to be a beneficial supplement for pregnant women, especially those who are at risk of developing anaemia. However, it is important to note that pregnant women should always consult their healthcare provider before taking any supplements. We selected eight articles [11,17,18,19,20,21,22,23] according to the inclusion criteria in this review. One [23] of these is explained in the next section as it is concerned with the study of pregnancy and breastfeeding. In South Sulawesi Province (Indonesia) [18], the study, focusing on pregnant women between 29 and 31 weeks of gestation, compared the efficacy of taking four capsules/day (each containing 500 mg of M. oleifera leaves powder (MOLP)) versus four capsules/day (each containing 60 mg and 400 µg of elemental iron and folic acid (IFA), respectively). It was demonstrated that using the first resulted in a significant improvement in their hemoglobin levels and led to a decrease in their perceived stress and cortisol levels. Furthermore, the newborns of mothers who received MOLP supplements had a higher birth weight when compared to those who were given IFA capsules. In the coastal area of Makassar (Indonesia), a randomized double-blind controlled design, pretest–posttest controlled, was investigated by Nadimin et al. [17] for three months. *Moringa* leaf extract (2 capsules × 800 mg) were contrasted versus IFA (60 mg iron and 250 µg folic) supplements to evaluate nutritional status on the basis of the weight gain and mid–upper arm circumference (MUAC) of non-anaemic women at 5–6 months of gestational age. Based on the results of this study, it appears that, for both studied groups, the intake levels of vitamins C and E, iron, and zinc fell below the recommended minimum adequacy of less than 70% of the recommended dietary allowance (RDA) and that there there was a significant change in the MUAC. Furthermore, the weight gain was greater in the IFA group (6.09 kg) compared to MOLP group (5.07 kg) during three months of intervention. Based on the results of this study, it appears that the intake levels of vitamins C and E, iron, and zinc fall below the recommended minimum adequacy of less than 70% of the recommended dietary allowance (RDA) [24]. In the area of the Puskesmas Tibawa (Tibawa Subdistrict, Gorontalo Regency, Indonesia), the study [18] suggests that *Moringa* extract may be effective in improving haemoglobin (Hb) levels in pregnant women. After six weeks of treatment, a majority of women in the *Moringa* group (54%) had an increase in HB values between 0.1–1.0 g/dL, and a substantial percentage (45%) recorded an increase in HB values between 1.5–2 g/dL. In contrast, the control group had a lower percentage of women with an increase in HB values between 0.1–1.0 g/dL (22%), and a majority (77%) that underwent no change in HB values. It is important to note that this study used a *Moringa* extract, rather than MOLP or whole-*Moringa* leaf powder, and compared the results to those from an FIA supplement. Manggul et al. [21] studied the use of *Moringa* leaf flour biscuit (cookies), which contained 78.3 kcal, 1.68 g, 4.63 g, 8.17 g and 0.99 mg of energy, protein, fat, carbohydrate and iron, respectively, for each biscuit. These authors used a design where the control (tablet of iron dose 2 times 250 mg) and intervention (*Moringa* leaf flour biscuit with dose 2 pieces biscuits in a day which that have *Moringa* flour content is 2.8 g each chip biscuit and dose of Fe tablets 2 times 250 mg) groups are not randomly selected. According to their findings, there was no significant effect of hemoglobin level on the intake of iron and zinc among the pregnant women studied in both the first and third trimesters. In a similar study by Loa et al. [22], the same recipe was used to prepare the biscuits. They studied the consumption of the intervention group, who were given *Moringa* leaf flour biscuit with a content of 2.8 g of *Moringa* leaf flour at a rate of 2 pieces/day and a Fe tablet 2 × 250 mg, and the control group, which received tablets of Fe 2 × 250 mg/day. The researchers evaluated the MCH, MCHC, and MCV values before and after a 60-day intervention period. The study group showed an increase in MCH values, while no significant difference was recorded in the control group. MCHC values varied in both groups, but the study group recorded a more significant increase. The MCV values increased significantly in the group that received *Moringa* biscuits, while the increase was not statistically significant in the control group. On the other hand, Andira et al. [19] conducted a study in which *Moringa* was used as an extract combined with royal jelly to evaluate its effect on hematocrit levels. The study compared the combination with a placebo over a period of 60 days. All three groups showed an increase in hematocrit values, but the group receiving the combination of *Moringa* extract and royal jelly showed the highest values. Basri et al. [11] studied the effect of powder *Moringa* (500 mg), extract *Moringa* (500 mg) and IFA (60 mg of Fe+ 200 µg of folic acid), this last sample being this last used as the control. Their multivariate analysis indicated that the use of *Moringa* extract significantly reduced the occurrence of stunted growth (*p* < 0.005), in children aged 36 to 42 months, as compared to other two groups (powder *Moringa* and IFA) and helped as a protective factor by decreasing the incidence of stunted growth by 0.431 times.

### 3.2. Moringa and Breastfeeding

Numerous studies have investigated the galactagogue properties of *Moringa*, which refer to its ability to initiate, maintain, and/or increase breast milk production. *Moringa* has been included into lactation-promoting products and it is listed on the National Institutes of Health LactMed Lactation Database [25]. Galactagogues are substances or medications that are believed to have such benefits. *Moringa* contains flavonoids and polyphenols, which are thought to contribute to its effect on two hormones (prolactin and oxytocin) that are associated with increased milk production. In accordance with the inclusion criteria of this review, we selected five articles [26,27,28,29,30] for this section. Pujiastuti et al. [28] utilized *Moringa* to produce biscuits that were given to women in their intervention group in their study. The biscuits were administered 24 h after delivery and combined with iron and vitamin A supplementation in the intervention group, while the control group only received iron and vitamin A. The children’s anthropometric parameters were measured before the intervention, on day 7, and day 14. The administration of *Moringa* biscuits had a positive impact on the children’s growth, as a greater weight gain was observed in the study group as early as day 7. These positive effects could be attributed to the presence of flavonoids, polyphenols, protein, potassium, and magnesium, which are nutrients that can contribute to increased milk production. Specifically, polyphenols stimulate the hormone oxytocin, which plays a vital role in stimulating the secretion of breast milk. Manganese stimulates the pituitary gland and induces the secretion of prolactin, which increases milk production, while potassium stimulates the secretion of oxytocin, which is necessary for the expulsion of milk from the mammary gland. In 2020, Sari et al. [23] conducted a study to investigate the impact of an intervention involving *Moringa* leaf extract (MLE), *Moringa* leaf powder (MLP), and IFA on the levels of docosahexaenoic acid (DHA) and arachidonic acid (AA) in breast milk. The lipid composition of breast milk is known to be sensitive to changes in the mother’s diet [31,32]. Sari et al. [23] specifically focused on two categories of lipids that are crucial for the development of an infant’s brain functions. The intervention lasted for 30 days, and the concentrations of DHA and AA in breast milk were measured in the three groups after the nutritional intervention. No significant differences were found in the concentrations of DHA and AA between the groups, although the levels of DHA in the MLE group were found to be more stable than those in the other two groups. The study also evaluated the quality and quantity of milk produced by monitoring growth during the first six months. In a study conducted by Zakaria et al. [29], a lactation education course was offered to all women included in the study. The study aimed to compare the effectiveness of two different *Moringa* supplements, namely, MLE capsules (3.2 g/day) in the intervention group and MLP capsules (3.2 g/day) in the control group, over a period of approximately 3 months. At the beginning of the study, there was no significant difference in the birth weight and height of the children in the two groups. After three months of treatment, the weight of male children showed no statistically significant difference, while the weight of female children in the intervention group was slightly higher. The height of the children was slightly higher in the control group at the end of the intervention, but the difference was not statistically significant. Based on the average of Z score of body length for age (BL/A) of infants, the control group had better growth, while the intervention group had better values based on the infant’s weight/body length. At the end of the intervention, values of the Z score of body mass index for age (BMI/A) had improved in both groups, with the intervention group showing better values, but the difference was not statistically significant. However, in the control group, the difference in the mean deviation of the infant’s BMI/A Z score was statistically significant before and after the intervention. The results of the study suggest that the administration of MLE capsules for three months may be more effective in preventing stunting compared to the administration of MLP. Hastuti et al. [27] carried out a longitudinal study to study effects of *M. oleifera*, folic acid, and iron supplementation on child development. The study included pregnant and breastfeeding mothers, and their children were monitored from 18 to 23 months of age in Jeneponto District (Indonesia). The sample size was 344 children, who were divided into three groups based on the type of supplementation received: MLP, MLE and IFA. Child development was assessed using the Denver developmental screening Ttst [27]. The authors obtained that 91.5% of children in the MLP group had normal social–personal development, which was higher than the 86.6% in the IFA group and 88.0% in the MLE group. Differences were also found in the social–personal development of children who were suspected to have developmental issues, with more children in IFA group than in the MLP or MLE groups displaying normal personal development. Overall, the study found no significant difference in children’s social–personal development between the three study groups. Suhartatik et al. [26] conducted a study to investigate the relationship between the administration of *Moringa* to mothers and the incidence of morbidity in infants during the first 6 months of their lives. The intervention group in the study received *Moringa oleifera* flour capsules, while the control group received iron folate capsules. The babies’ health status was monitored every three months, and the results showed that taking *Moringa* supplements is significantly better for babies during the first 3 months of life compared to taking iron tablets. Furthermore, the study found that the benefits of taking *Moringa* supplements are even greater in the long term as *Moringa* leaf powder is 7857 times more effective in reducing children’s morbidity during the following 3 months compared to iron tablets.

## 4. Discussion

*M. oleifera* is an abundant and nutrient-rich local food source that is widely underutilized in low-income countries. It is commercially grown in various parts of the world, including India, Africa, South and Central America, Mexico, Hawaii, and throughout Asia and Southeast Asia. *M. oleifera* belongs to the *Moringaceae* family and is extensively studied and used for both human and animal applications. This plant is drought-resistant and can thrive in various soil types and wet or dry areas. Almost every part of the plant is potentially useful, including the seeds, leaves, flowers, and pods, with each part being consumed for nutritional purposes. The leaves of the *Moringa* plant are particularly nutrient-dense and contain a significant number of essential amino acids, vitamins, and minerals, including calcium, iron, potassium, magnesium, and zinc. They are also rich in vitamins A, B complex vitamins (B1, B2, B3), C, and E, and contain 17 fatty acids, including three polyunsaturated fatty acids (PUFA) [33]. The leaves are the most commonly consumed part of the plant for nutritional purposes and contain various bioactive compounds. *Moringa* is also recognized as an herbal galactagogue due to its high content of macro- and micronutrients that stimulate milk production in lactating mothers [34]. It has been widely studied [28,30,35,36,37] for its galactogenic properties, and scientific evidence proves its positive effect on the production of breast milk, supporting the growth of newborns. *Moringa* is considered to be a nutritious food supplement that can save human lives, particularly in countries with poor nutrition. The studies included in this paper present *Moringa* as a food supplement in its various forms, including in dried leaves, as a leaf extract or ingredient in food preparations, or combined with other supplements.

Several studies [18,22,38,39] have suggested that *Moringa* supplementation can effectively prevent anaemi as it contains sufficient levels of iron and has similar preventive effects in commonly used folic iron supplements during pregnancy. *Moringa* has shown good in vitro bioaccessibility and bioavailability of iron [40], although studies demonstrating its in vivo bioavailability are not available. Due to its high iron content, most of the studies available in the literature on the effects of *Moringa* as a dietary supplement during pregnancy focus on anaemia. Iron deficiency anemia (IDA) is a prevalent health issue during pregnancy, especially in developing countries. The main reasons for the higher prevalence of iron deficiency anemia (IDA) during pregnancy in low-income countries are: inadequate intake of iron-rich foods, lack of proper sanitation practices, access to unsafe drinking water, iron depletion caused by parasites (such as malaria or intestinal worms), anemia among adolescents, and the occurrence of teenage and repeated pregnancies. These factors contribute to a significant increase in the prevalence of IDA in pregnancy in these nations [15]. In addition, *Moringa* leaves are a rich source of nutrients that are essential for the growth and development of the foetus, as well as for maintaining the nutritional status of pregnant women, according to a study by Nadimin et al. [17]. In this study, the authors suggest that *Moringa* supplementation can improve maternal, fetal, and infant outcomes, making it a beneficial supplement during pregnancy and lactation, especially in low- and middle-income settings. During the critical period of pregnancy and postpartum, nutrition plays a pivotal role in ensuring optimal health for both the mother and the child. Failing to provide sufficient macronutrients or micronutrients during this time can result in lifelong effects on neurodevelopment, as illustrated the study of King et al. [35]. Therefore, optimizing the nutritional intake of both the mother and baby is crucial to improving maternal and infant health outcomes. Pregnancy and the postpartum period impose increased energy and nutrient requirements that are vital for optimal growth and development. Inadequate fulfillment of these nutritional needs, even by small amounts, can have irreversible negative impacts on the health of both the mother and the baby. Thus, supplementing with micronutrients during pregnancy and lactation is widely accepted, especially in developing countries where malnutrition is prevalent and has a detrimental impact on the health and future of entire communities. Micronutrient deficiencies occur when there is an inadequate intake of essential vitamins and minerals from the diet to meet the recommended daily allowances required for proper health, growth, and development, as defined WHO [41]. Ensuring adequate micronutrient intake for pregnant and lactating mothers is crucial to maintaining optimal health for both mother and child. Micronutrient supplementation during this period can help to prevent deficiencies that can have long-term negative impacts on the mother’s health, as well as her ability to care for herself and her child. Such deficiencies pose a significant threat to the health and well-being of women of reproductive age and their children, and without intervention, they can perpetuate the cycle of malnutrition for generations to come. Inadequate nutrition during pregnancy can hinder the growth and development of the fetus and increase the risk of chronic diseases and other conditions later in life. Poor maternal nutrition has also been linked to stunted growth, nutrition-related chronic diseases, reduced educational and income achievements, and even decreased birth weight in the next generation. It is, therefore, critical to recognize the importance of proper nutrition during pregnancy and lactation to ensure the long-term health and well-being of both the mother and child [42,43]. Ensuring global nutrition and food security is a critical challenge that must be addressed by the 2030 Agenda. One approach to achieving this goal involves exploring alternative food sources, especially in low-resource settings where sustainable projects can be developed. Discovering new, nutrient-rich foods that are produced sustainably can ensure regular access to vital nutrients for people. Utilizing unconventional food plants, such as indigenous and wild plant species, can enhance food quality and security in multiple ways. This approach can also contribute to achieving several of the Sustainable Development Goals, including promoting sustainable agriculture, improving health and well-being, supporting responsible production and consumption, and protecting the environment [44]. Stohs and Hartman’s review [6] found that oral ingestion of *M. oleifera* leaves and leaf extracts at achievable doses did not produce adverse effects in any human or in vitro studies, or when animal studies were extrapolated to humans. The studies reviewed suggest that *Moringa* has several positive effects on various health variables during pregnancy and lactation. *Moringa* leaves possess a rich nutritional composition and have shown potential in increasing breastmilk production, indicating their possible use as a supplement in populations with high rates of malnutrition and micronutrient deficiencies. Furthermore, *Moringa* can serve as a valuable resource in regions which are vulnerable to climate change. *Moringa* is resilient to drought, grows rapidly and easily, and yields multiple harvests of leaves and edible seed pods throughout the year. These nutrient-dense leaves can be dried and powdered, providing a portable, shelf-stable food source year-round. Due to its ease of cultivation, nutrient density, antioxidant properties, and potential galactagogue effect, further research is needed to explore the use of moringa leaf powder as a potential tool at the household or community level to improve childhood and maternal undernutrition [9]. Nonetheless, the evidence indicates that *Moringa* administration has measurable beneficial effects that are comparable to those of iron and folic acid supplementation. Since these minerals are often deficient in vulnerable situations and food insecurity and play crucial roles during pregnancy and lactation, further studies are necessary to determine dose-dependent benefits, establish the treatment duration required to identify measurable benefits, and develop a useful protocol for standardizing the use of this valuable herb as a dietary supplement during pregnancy and lactation.

The limitations of this review include that it assesses the potential effects of *Moringa* as a dietary supplement during pregnancy and the postpartum period. All the trials included in this review were conducted in South East Asia (Indonesia and the Philippines). The populations in this geographical region are predominantly of the same or closely related races. However, other races in Africa and South America, where the plant is naturally widespread and abundant, have different anthropological characteristics. To generalize the concept and conclusions, it would be beneficial to include populations from other low- and middle-income countries (LMICs) in further studies. Another limitation of this review is the lack of detailed data on the doses administered and the duration of treatment for each intervention considered, which prevents a precise definition of the effects of the supplement in relation to dosage.

## 5. Conclusions

*Moringa* appears to be a useful local food to prevent nutritional deficiencies at this specific physiological stage. Further studies are needed to monitor its dose-dependent effects and to assess its possible adverse effects.

## Data Availability

The data presented in this study are available on request from the corresponding author.
